# Gastrocnemius Echo Intensity Is Associated with Walking Distance and Hemodynamic Improvements After Endovascular Therapy in Lower Extremity Artery Disease

**DOI:** 10.3390/jcm14228189

**Published:** 2025-11-18

**Authors:** Satoshi Yuguchi, Yusuke Ochi, Yukari Sagata, Mitsuhiro Idesako, Shino Maeda, Masahito Taniguchi

**Affiliations:** 1 Department of Physical Therapy, School of Health Sciences, Toyohashi SOZO University, Aichi 440-8511, Japan; 2 Department of Rehabilitation, Fukuyama Cardiovascular Hospital, Hiroshima 720-0804, Japan; 3 Department of Cardiology, Fukuyama Cardiovascular Hospital, Hiroshima 720-0804, Japan

**Keywords:** lower extremity artery disease, echo intensity, walking distance

## Abstract

**Background**: This study aimed to validate the associations between the echo intensity (EI) of the gastrocnemius muscle before endovascular therapy (EVT) and post-EVT changes in the 6-min walk distance (6MWD), ankle–brachial index (ABI), and other skeletal muscle indicators in patients with lower extremity artery disease (LEAD). **Methods**: A total of 29 male patients (mean age: 73.5 years) presenting with Fontaine stage II LEAD participated in this research. The EI of the gastrocnemius muscle before EVT was assessed using ultrasonography. Based on the tertiles of EI values, participants were categorized into low (*n* = 10), mid (*n* = 10), and high (*n* = 9) groups. The ABI, gastrocnemius thickness, EI, and 6MWD were examined before EVT, at discharge, and at 6 months after EVT. Both cross-sectional and longitudinal comparisons were conducted across the three groups before and after EVT. **Results**: Before EVT, the high group demonstrated lower gastrocnemius thickness (9.0 vs. 12.7 mm, *p* < 0.01) and shorter 6MWD (258 vs. 391 m, *p* < 0.05) than the low group. At 6 months after EVT, the high group demonstrated lower ABI than the low and mid groups. The low (from 391 to 467 m, *p* < 0.05) and mid (from 375 to 416 m, *p* < 0.05) groups exhibited improvements in 6MWD. However, the high group did not (from 258 to 312 m, *p* = 0.1). **Conclusions**: EI before EVT was associated with improvements in ABI and 6MWD in patients with LEAD after EVT.

## 1. Introduction

Recently, the proportion of patients with lower extremity artery disease (LEAD) has been increasing [1]. LEAD presents as either asymptomatic disease or intermittent claudication, which makes walking difficult due to leg pain triggered by walking long distances. According to the TransAtlantic Inter-Society Consensus II, the mortality rate of LEAD in patients with intermittent claudication is 2.5 times higher than that in patients without intermittent claudication. Furthermore, cardiovascular-related adverse events are the leading cause of death [1]. Exercise therapy is effective in improving intermittent claudication symptoms. However, if intermittent claudication does not improve or if, according to the location of the stenotic lesion, revascularization is indicated, revascularization may be performed to enhance lower-limb blood flow. Although revascularization improved walking distance, improvements in walking distance were minimal compared with those in lower-limb blood flow [2]. Based on this finding, the achievement of a greater walking distance cannot be explained by improvement in lower-limb blood flow alone. In addition, walking distance is related to lower-limb blood flow, inflammation, vascular endothelial function, and skeletal muscle metabolism [3]. According to previous reports on skeletal muscle, myopathy or neuropathy due to lower-limb ischemia is present in the skeletal muscles of the lower extremities in patients with LEAD [4]. A previous study using computed tomography (CT) showed that lower limbs with ischemia had a smaller lower-leg muscle area and higher intramuscular fat percentages compared with those without ischemia. Moreover, intramuscular fat percentage was correlated with 6-min walk distance (6MWD) [5,6]. In light of the abovementioned reports, it may be necessary to focus on intramuscular fat percentage in the lower extremity to identify factors influencing improvements in walking distance after revascularization. However, frequent evaluation of intramuscular fat using a CT scan is challenging. Furthermore, the procedure is also associated with radiation exposure.

Meanwhile, based on recent studies, echo intensity (EI) measured using ultrasonography can easily and noninvasively evaluate intramuscular fat percentage [7]. In particular, it has a strong positive correlation with intramuscular fat percentage obtained via muscle biopsy, and skeletal muscle with myopathy or neuropathy shows higher EI values [8].

Patients with LEAD have skeletal muscle abnormalities, such as myopathy and neuropathy, due to leg ischemia, according to previous reports. However, the impact of these abnormalities on improvements in walking distance after revascularization remains unknown. We hypothesized that the EI of skeletal muscle before revascularization is related to improvements in walking distance after revascularization and that EI can be a predictor of improvements in walking distance after revascularization. Therefore, EI can be an indicator of any adaptations of treatment interventions in patients with LEAD.

The current study aimed to investigate the association between the EI of the gastrocnemius muscle before endovascular therapy (EVT) and changes in walking distance after EVT in patients with LEAD who present with intermittent claudication.

## 2. Materials and Methods

### 2.1. Participants

A total of 29 male patients with Fontaine stage II LEAD, with an average age of 73.5 years, underwent EVT between March 2020 and August 2023. The patients were assessed before EVT, at discharge, and at 6 months after EVT. Patients who met any of the following criteria were excluded from the analysis: being female, use of a walking aid, unstable walking due to stroke or orthopedic disease, or unstable general condition due to heart failure or other diseases. This study was performed in accordance with the Declaration of Helsinki and was approved by the Ethics Committee of Fukuyama Cardiovascular Hospital (approval number 59).

### 2.2. Study Design

This prospective cohort study compared pre- and post-EVT outcomes over a 6-month period. The EI of the gastrocnemius muscles in both legs was measured using ultrasonography before EVT. Based on the tertiles of the EI values in the leg with a lower ankle–brachial index (ABI), the participants were classified into the low, mid, and high groups. Data on age, body mass index (BMI), medical history, presence or absence of bilateral lesions, parts of arterial lesions, and ABI were obtained from the medical records. The 6MWD, ABI, gastrocnemius thickness (GT), and EI were measured via ultrasonography 1 day before EVT, at discharge after EVT, and at 6 months after EVT. Normal gait speed, grip strength, and skeletal muscle mass index (SMI) were examined using bioelectrical impedance analysis on the day before EVT and at 6 months after EVT.

The primary outcome was the 6MWD at 6 months after EVT. Secondary outcomes were the changes in 6MWD, ABI, and physical performance measures.

#### 2.2.1. Assessment of Gastrocnemius Thickness (GT) and Echo Intensity (EI)

**Figure 1:**
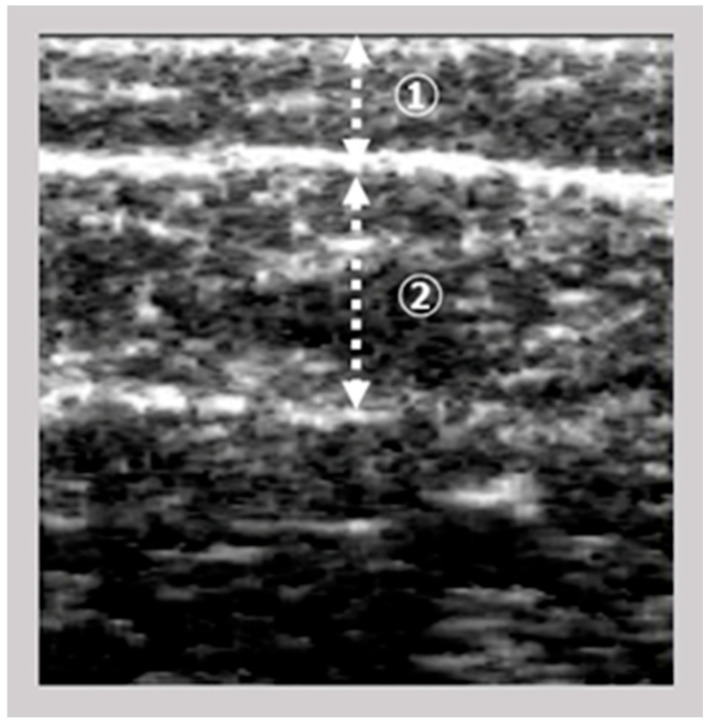
Ultrasonographic image of the gastrocnemius muscle; ① subcutaneous fat thickness; ② gastrocnemius thickness (GT).

The images of the gastrocnemius muscle were obtained using an ultrasound device (View’s i; SAKAI Medical Science Co., Ltd., Tokyo, Japan) equipped with a 6-MHz linear array probe. In all patients, ultrasonography measurements were performed under fixed settings, including B-mode, a fixed dB dynamic range, fixed gain, and fixed depth of focus, which were preconfigured for skeletal muscle imaging and intentionally made non-modifiable by the manufacturer. The mode settings are not disclosed due to the device’s patent protection. The participants were assessed in a seated position with both knees flexed at 90° and the ankles at 0°. The examiner vertically and gently positioned the probe on the right medial gastrocnemius at the point of maximum below-knee circumference. The A-mode display on the device was monitored simultaneously. The examiner captured images of the subcutaneous adipose tissue and gastrocnemius muscle (Figure 1). The subcutaneous fat thickness (SFT) was defined as the distance between the skin surface and the upper fascia of the gastrocnemius. Meanwhile, the GT was defined as the distance between the subcutaneous fascia and the deep fascia. A previous study investigated the intra-rater reliability of GT measurements in healthy adults when the probe pressure was maintained below 100 gf [9] reported decreased reliability under this condition. Based on these results, we set the probe pressure at 200 gf in this study to minimize measurement error.

**Figure 2:**
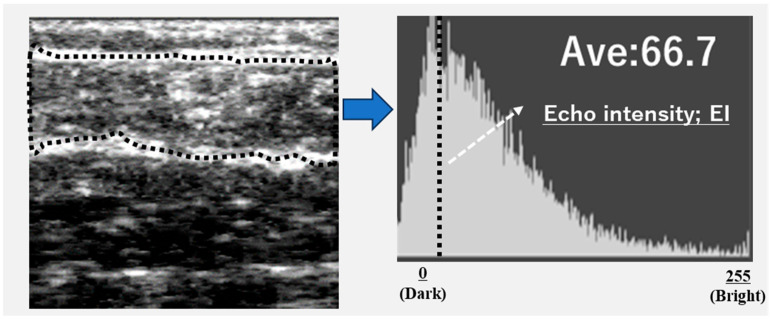
Calculation of echo intensity from the ultrasonographic image.

Ultrasound images were exported as Joint Photographic Experts Group (JPEG) files, and the EI of the gastrocnemius muscle was calculated using Adobe Photoshop Elements (Adobe Systems, Inc., San Jose, CA, USA). The target area was selected to include as much muscle tissue as possible while avoiding the surrounding fascia. The selected area was converted to an 8-bit grayscale image, with the mean image brightness presented as a value ranging from 0 (black) to 255 (white) (Figure 2). The EI was calculated as the mean image brightness [10]. This study analyzed the EI and GT of the leg with the lower ABI.

One specialized and well-trained examiner performed ultrasonography assessments. The repeatability of GT and EI measurements was assessed using the intraclass correlation coefficient (ICC). The ICC employed models (1.1) and (1.2) for GT and EI. The ICC (1.1) for GT and EI was 0.98 (95% confidence interval [CI]: 0.96–0.99) and 0.97 (95% CI: 0.93–0.99), whereas the ICC (1.2) for GT and EI was 0.99 (95% CI: 0.98–0.99) and 0.98 (95% CI: 0.96–0.99), respectively.

#### 2.2.2. Evaluation of 6MWD

The participants walked back and forth along a 30-m hospital corridor. Before the 6MWD test, the participants were instructed to walk as far as possible within 6 min. They were informed that they could take breaks if they experienced significant leg pain due to lower extremity ischemia and could resume walking once the pain subsided. In this study, none of the participants were unable to complete the 6MWD test due to adverse events, such as dyspnea and cardiovascular-related complications. 6MWD was measured once following the American Thoracic Society (ATS) guidelines. Assessors were blinded to the baseline results and EI classification to minimize potential bias.

#### 2.2.3. Investigation of Skeletal Muscle Mass Index, Grip Strength, and Gait Speed

The SMI was assessed using a bioelectrical impedance device (In-Body S10, InBody Japan Corp., Tokyo, Japan). The participants were placed in the supine position. The appendicular skeletal muscle mass (kg) of all participants was measured. SMI was calculated by dividing the muscle mass by height in meters squared (kg/m^2^).

Grip strength was assessed using the Jamar dynamometer (NC70142, North Coast Medical, Inc., California, USA) and was defined as the highest value obtained from the right or left hand across trials. Walking speed (m/s) was measured once and was defined as the time taken to walk a 5-m distance at a comfortable pace.

### 2.3. Statistical Analysis

**Figure 3:**
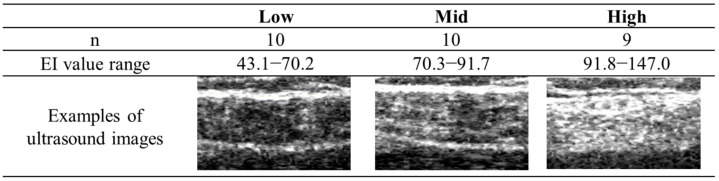
Range of echo intensity (EI) values and examples of ultrasound images for each group, divided into tertiles.

The Shapiro–Wilk test was performed on all data within each group to assess normality prior to data analysis. Twenty-nine participants were classified into the three groups based on the tertiles of the EI values in the leg with a lower ABI, as follows: low (*n* = 10, range: 43.1–70.2), mid (*n* = 10, range: 70.3–91.7), and high (*n* = 9, range: 91.8–147.0) groups (see Figure 3). The characteristics of the participants were compared using one-way analysis of variance (ANOVA) or the χ^2^ test. Cross-sectional comparisons of the data before EVT, at discharge, and at 6 months after EVT among the three groups were conducted using ANOVA or the Kruskal–Wallis test. If significant differences were found, multiple comparison tests were performed using Tukey’s test or Bonferroni’s correction.

Longitudinal comparisons of 6MWD, ABI, EI, and GT of the lower ABI leg in each group before EVT, at discharge, and at 6 months after EVT were performed using repeated-measures ANOVA or the Friedman test. If significant differences were found, a multiple comparison test using Bonferroni’s correction was conducted. The SMI, grip strength, and gait speed of each group before EVT and at 6 months after EVT were compared using the paired *t*-test. Statistical analyses were performed using IBM SPSS Statistics Version 27 (IBM Corp., Armonk, NY, USA), and a two-tailed *p* value of <0.05 was considered statistically significant.

## 3. Results

Table 1 shows that the low, mid, and high groups demonstrated no significant differences in patient characteristics. However, the high group exhibited a higher average age and a lower BMI than the low and mid groups. Concerning the lesion site, none of the participants had lesions in the below-knee artery area. A higher proportion of participants in the low group were more likely to present with lesions limited to the iliac artery. Meanwhile, the high group consisted of a greater proportion of participants with lesions that involved both the iliac and femoropopliteal arteries. The lower ABI values in the high group did not significantly differ from those in the low and mid groups. However, the mean higher ABI value remained less than 0.9.

Table 2 shows the cross-sectional comparisons of the 6MWD, ABI, GT, EI, physical performance, and SMI before EVT, at discharge, and at 6 months after EVT. The high group demonstrated a significantly lower 6MWD than the low (*p* < 0.05) and mid groups (*p* < 0.05) before EVT, at discharge, and at 6 months after EVT based on ANOVA and Tukey’s test. However, no significant differences in the 6MWD were observed between the low and mid groups. Before EVT, there was no significant difference in the ABI among the three groups using ANOVA and Tukey’s test. At discharge, there were no significant differences among the three groups using ANOVA and Tukey’s test. However, at 6 months after EVT, the high group demonstrated a significantly lower ABI than the low group (*p* < 0.05) using ANOVA and Tukey’s test. The ABI did not increase by more than 0.9. Before EVT, at discharge, and at 6 months after EVT, the high group demonstrated a significantly lower GT (*p* < 0.01) and a higher EI (*p* < 0.01) than the low group using the Kruskal–Wallis test and Bonferroni’s correction. Furthermore, at 6 months after EVT, the high group demonstrated a significantly lower GT (*p* < 0.01) and a higher EI (*p* < 0.01) than the mid group. The EI of the mid group was significantly higher than that of the low group (*p* < 0.05) before EVT. However, at discharge and 6 months after EVT, the EI did not significantly differ between the mid and low groups. The high group exhibited a significantly lower gait speed (*p* < 0.05) and lower grip strength (*p* < 0.05) than the low group before EVT and at 6 months after EVT using ANOVA and Tukey’s test.

**Figure 4:**
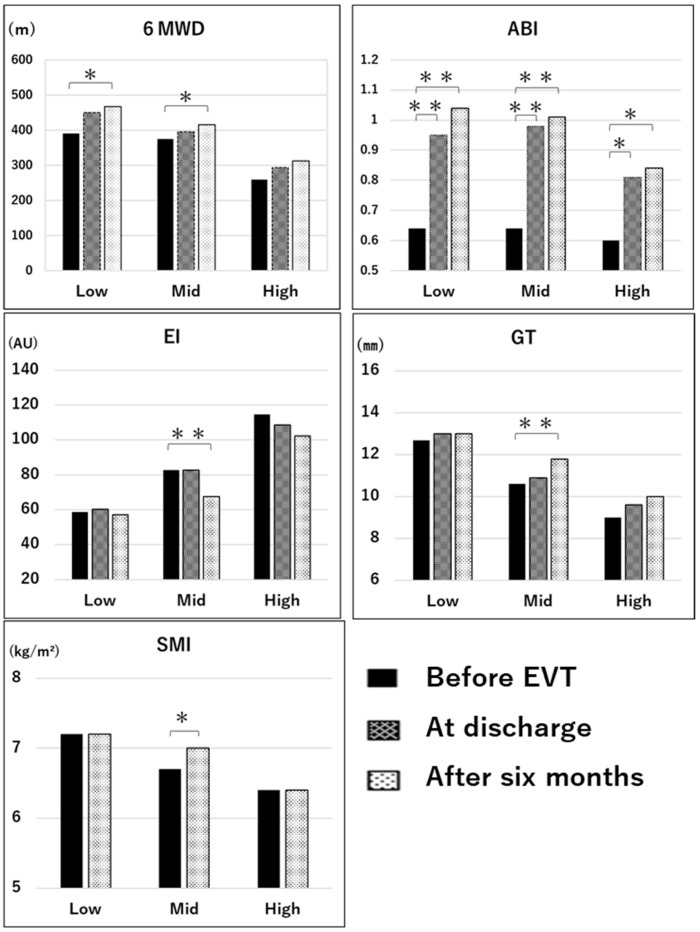
Longitudinal comparisons of each parameter in the three groups between before EVT, at discharge, and six months after EVT. *, vs. Low *p* < 0.05; **, vs. Low *p* < 0.01.

Figure 4 shows the longitudinal comparisons of each parameter in the low, mid, and high groups. There were significant improvements in the 6MWD at 6 months after EVT in the low (*p* < 0.05, effect size = 0.96, power = 0.99) and mid groups (*p* < 0.05, effect size = 0.78, power = 0.99) compared with values before EVT using repeated-measures ANOVA and the multiple comparison of Bonferroni’s correction. However, there was no significant improvement in the 6MWD in the high group based on repeated-measures ANOVA (*p* = 0.13, effect size = 0.56, power = 0.88). There were significant improvements in the ABI in the low (*p* < 0.01, effect size = 2.21, power = 1.0), mid (*p* < 0.01, effect size = 2.13, power = 1.0), and high groups (*p* < 0.05, effect size = 0.7, power = 0.98) at 6 months after EVT compared with before EVT, and it exceeded 1.0 in the mid and low groups. However, the ABI in the high group remained below 0.9 even at 6 months after EVT. There were significant differences in GT (*p* < 0.01, effect size = 0.61, power = 0.80), EI (*p* < 0.01, effect size = 0.47, power = 0.56) based on the Friedman test and the multiple comparison test of Bonferroni’s correction, and in SMI (*p* < 0.05, effect size = 0.26, power = 0.11) using the paired *t*-test in the mid group before EVT compared with 6 months after EVT. The GT, EI, and SMI did not differ between the low and high groups (effect size = 0.09–0.21, power = 0.07–0.15). The gait speed and grip strength did not significantly change before and after EVT among the three groups.

## 4. Discussion

Previous studies have not reported any associations between baseline EI, stratified into tertiles, and improvements in walking distance or lower-limb hemodynamics in patients with LEAD after EVT. This study is the first to demonstrate that preoperative EI of the gastrocnemius muscle, as an intramuscular fat content indicator, is associated with improvements in 6MWD and lower-limb hemodynamics in these patients.

The high group demonstrated a significantly lower 6MWD, GT, gait speed, and grip strength than the low group before EVT, at discharge, and at 6 months after EVT. Increased intramuscular fat percentage is observed in skeletal muscles with myopathy or neuropathy. In addition, EI, as measured using ultrasonography, increases in patients with neuromuscular diseases presenting with myopathy or neuropathy [11]. Moreover, previous studies have reported factors associated with EI. Healthy adults who are older demonstrate higher EI values, and women have higher EI values than men [7]. Regarding physical performance, the EI of the quadriceps muscles is independently associated with knee extensor strength after adjusting for confounders such as age, height, weight, and SFT [12]. In addition, muscle strength is more strongly associated with EI than with muscle thickness, and the EI of patients with sarcopenia is higher than that of patients without sarcopenia [10]. Meanwhile, in our previous study, the median EI value of the gastrocnemius muscles in healthy adults with an average age of 73.2 years was 72.0 [13]. In this study, the average age of patients with LEAD was 73.5 years, and the median EI values before EVT were 58.7 in the low group, 82.9 in the mid group, and 114.6 in the high group, respectively. Based on a previous report showing that patients with LEAD have increased intramuscular fat percentages due to myopathy or neuropathy [5], the high group might have more severe myopathy or neuropathy due to lower-limb ischemia than the low and mid groups. Therefore, the high group, with the highest EI values, had a lower 6MWD and poorer physical performance from before to 6 months after EVT, compared with the low and mid groups.

The low and mid groups showed significant improvements in the 6MWD. In addition, only the mid group exhibited significant improvements in skeletal muscle quantity and quality, as indicated by changes in EI, GT, and SMI from before EVT to 6 months after EVT. By contrast, the high group had a significant increase only in the lower-limb blood flow, as reflected by changes in ABI from before to after EVT. Before EVT, the low group had better ABI, EI, GT, and SMI values compared with the other groups. In particular, the low group had a lower median EI than the age-matched healthy adults (58.7 vs. 72.0), and their average SMI was higher than 7.0 kg/m^2^, which is defined as the cutoff value for decreased skeletal muscle mass [14]. The low group did not have myopathy or neuropathy due to lower-limb ischemia, and the limitation of walking distance may have been caused by the limitation of lower-limb blood flow alone. Therefore, the 6MWD in the low group significantly increased due to improvements in blood flow after EVT. Similarly, the median EI value of the mid group before EVT was slightly higher than that of age-matched healthy adults, and the GT in the mid group was lower than that in the low group. Furthermore, considering that an average SMI of 6.7 kg/m^2^ was slightly below the cutoff value of 7.0 kg/m^2^, the mid group might have presented with mild myopathy in the gastrocnemius muscles before EVT. Considering that intramuscular fat percentage is an independent factor influencing 6MWD [5], the limitation in walking distance is likely attributed to both myopathy and lower-limb ischemia. Thus, before EVT, the mid group had a slightly lower 6MWD than the low group. After EVT, the ABI and 6MWD of the mid group significantly improved to the same level as those of the low group. In addition, the mid group also exhibited improvements in EI, GT, and SMI. In light of the mild myopathy in the mid group, myopathy due to ischemia may have improved over the 6 months after EVT. Based on this finding, improvements in both lower-limb blood flow and myopathy contributed to the significant improvements in the 6MWD.

The ABI before EVT in the high group did not differ from that of the low and mid groups. However, the median EI value in the high group was significantly higher than that of age-matched healthy adults. Among the three groups, the high group demonstrated the lowest 6MWD, GT, and SMI. Thus, before EVT, the gastrocnemius muscle in the high group might have been accompanied by severe myopathy, and both severe myopathy and lower-limb ischemia might be factors influencing the limitation of the 6MWD test. Furthermore, the ABI in the high group significantly improved. However, the average ABI was below the normal value of 0.9 at 6 months after EVT. In the mid group, skeletal muscle abnormalities improved, accompanied by an increase in lower-limb blood flow. However, there were no significant improvements in EI, GT, and SMI in the high group, indicating that severe myopathy observed before EVT persisted for 6 months after EVT. Therefore, in the high group, the persistence of severe myopathy and mild lower-limb ischemia might have contributed to the lack of significant improvement in the 6MWD after EVT. Although lower-limb blood flow improved after EVT, severe myopathy did not improve within the preceding 6 months. Moreover, the long-term association between increases in lower-limb blood flow and skeletal muscle abnormalities remains unclear. Factors such as baseline physical performance, lesion complexity, and potential confounders may influence this relationship. Further studies are warranted to validate our findings regarding ABI improvement.

By contrast, improvements in lower-limb blood flow and the quantity and quality of skeletal muscle after EVT may be influenced by physical activity (PA) after discharge [15]. Furthermore, it has been reported that when the 6MWD is <400 m, mobility is impaired [16], and when the 6MWD is <300 m, the ability to go out is limited according to a Japanese study [17]. The average 6MWD at discharge was below 300 m only in the high group. This finding indicates that the high group might have a lower PA between discharge and 6 months after EVT compared with the other groups. A systematic review revealed that, after revascularization, patients with LEAD who underwent exercise therapy had a greater improvement in walking distance than those without exercise therapy. This combination also led to significant improvements in endothelial function and skeletal muscle metabolic function [18]. In the future, the effects of exercise and PA after EVT on improvements in skeletal muscle should be assessed using EI measured using ultrasonography.

This study has several limitations. First, since EI values depend on the settings of the ultrasound device, they cannot be compared when different settings are used. Second, this is an observational study in which participants were advised to perform only walking exercises and were not provided with exercise therapy or PA measurements, such as step counts, after EVT. Therefore, the participant’s PA after EVT is unclear. Third, this study included only 29 participants; thus, baseline ABI may have differed in the high EI group (power = 0.1). Moreover, the improvement in 6MWD after EVT in the high group may have been influenced by potential confounding factors, such as age and lesion site. Finally, the association between pre-EVT EI and post-EVT ABI improvement remains debatable; thus, further studies are warranted.

## 5. Conclusions

This study was an observational cohort study, and exercise therapy and post-discharge PA monitoring were not conducted. Therefore, the results of this study should be interpreted considering this limitation. The EI of the gastrocnemius muscle before EVT was associated with improvements in 6MWD and ABI after EVT. Hence, EI can be a useful indicator for assessing postoperative progress and determining the indications for therapeutic interventions.

## Figures and Tables

**Table 1:** Characteristics of patients with LEAD before EVT.

	Total (*n* = 29)	Low (*n* = 10)	Mid (*n* = 10)	High (*n* = 9)	P	F	χ^2^
Age; years (mean ± SD)	73.5 ± 8.7	69.9 ± 8.8	73.0 ± 10.4	77.9 ± 4.5	0.1	2.2	
BMI: kg/m^2^ (mean ± SD)	23.4 ± 3.4	24.8 ± 2.9	23.2 ± 3.6	22.0 ± 3.4	0.2	1.7	
Diabetes; *n* (%)	16(55.1)	6(60.0)	6(60.0)	4(44.4)	0.7		0.6
Hypertension; *n* (%)	25(86.2)	9(90.0)	7(70.0)	9(100)	0.2		3.8
Hyperlipidemia; *n* (%)	19(65.5)	7(70.0)	6(60.0)	6(66.6)	0.9		0.2
CKD; *n* (%)	4(13.8)	0(0)	3(30.0)	1(11.1)	0.2		3.9
CVD or CVA; *n* (%)	7(24.1)	3(30.0)	2(20.0)	2(22.2)	0.4		0.5
Orthopedic diseases; *n* (%)	5(17.2)	1(10.0)	2(20.0)	2(22.2)	0.8		0.6
Lesion site; *n* (%)							
Bilateral leg lesions	9(31.0)	2(20.0)	4(40.0)	3(33.3)	0.6		0.5
Iliac artery area alone	14(48.3)	7(70.0)	5(50.0)	2(22.2)	0.1		4.4
Iliac + femoropopliteal artery area	15(51.7)	3(30.0)	5(50.0)	7(77.7)	0.1		4.4
Below-knee artery area	0(0)	-	-	-	-		
ABI (mean ± SD)							
Higher ABI	0.9 ± 0.2	1.0 ± 0.2	0.9 ± 0.1	0.8 ± 0.2	0.2	1.5	
Lower ABI	0.6 ± 0.1	0.6 ± 0.2	0.6 ± 0.1	0.6 ± 0.2	0.8	0.3	

BMI, body mass index; CKD, chronic kidney disease; CVD, cardiovascular disease; CVA, cerebral vascular disease; ABI, ankle brachial index.

**Table 2:** Comparisons among three groups before EVT, at discharge, and six months after EVT.

		Low	Mid	High	Effect Size	Power
6MWD; m (mean ± SD)	Before EVT	391.0 ± 31.4	375.2 ± 25.2	258.8 ± 23.2 **☨	0.7	0.9
	AT discharge	450.4 ± 23.4	395.8 ± 21.3	294.2 ± 22.9 **☨	0.7	0.9
	After six months	466.8 ± 27.0	415.5 ± 21.9	312.8 ± 32.7 **☨	0.8	1.0
	Δ (Post–Pre)	75.8 ± 76.2	40.3 ± 41.0	54.0 ± 95.7	0.2	0.2
ABI (mean ± SD)	Before EVT	0.6 ± 0.1	0.6 ± 0.1	0.6 ± 0.1	0.1	0.1
	AT discharge	1.0 ± 0.1	1.0 ± 0.1	0.8 ± 0.1	0.5	0.6
	After six months	1.0 ± 0.1	1.0 ± 0.1	0.8 ± 0.1 *	0.6	0.8
GT; mm (median; range)	Δ (Post–Pre)	0.4 ± 0.2	0.4 ± 0.2	0.2 ± 0.2	0.4	0.4
	Before EVT	12.7 (10.6–18.1)	10.6 (9.3–13.2)	9.0 (7.8–11.7) **	0.9	0.9
	AT discharge	13 (10.1–17.9)	10.9 (9.3–13.7)	9.6 (8.1–11.4) **	0.9	0.9
	After six months	13 (11.4–17.2)	11.8 (10.4–15.6)	10 (7.65–10.9) **☨☨	0.9	0.9
EI; AU (median; range)	Before EVT	58.7 (43.1–70.2)	82.8 (70.6–91.7) *	114.6 (94.7–147.0) **☨	2.2	1.0
	AT discharge	60.4 (37.5–89.7)	82.8 (71.7–92.9)	108.5 (81.6–167.9) **	1.5	0.9
	After six months	57.3 (45.3–94.8)	67.6 (56.2–88.8)	102.2 (87.6–129.6) **☨☨	1.3	0.9
Gait speed; m/s (mean ± SD)	Before EVT	1.1 ± 0.2	1.0 ± 0.2	0.8 ± 0.1 *	0.5	0.5
	After six months	1.2 ± 0.3	1.0 ± 0.1	0.9 ± 0.2 *	0.5	0.6
Grip strength; kg (mean ± SD)	Before EVT	34.6 ± 5.0	30.8 ± 5.5	27.8 ± 5.1 *	0.6	0.7
	After six months	34.3 ± 5.3	31.8 ± 6.4	27.9 ± 4.1 *	0.5	0.7
SMI; kg/m^2^ (mean ± SD)	Before EVT	7.2 ± 0.7	6.7 ± 0.9	6.4 ± 0.6	0.5	0.5
	After six months	7.2 ± 0.8	7.0 ± 0.9	6.4 ± 0.6	0.5	0.7

6MWD, 6-min walking distance; ABI, ankle-brachial index; GT, gastrocnemius thickness; EI, echo intensity; SMI; skeletal muscle index. *, vs. Low *p* < 0.05; **, vs. Low *p* < 0.01; ☨, vs. Mid *p* < 0.05; ☨☨, vs. Mid *p* < 0.01; AU, arbitrary unit.

## Data Availability

The data presented in this study are not available in a more detailed form due to data protection reasons.
